# Mathematical Modeling Reveals That the Administration of EGF Can Promote the Elimination of Lymph Node Metastases by PD-1/PD-L1 Blockade

**DOI:** 10.3389/fbioe.2019.00104

**Published:** 2019-05-14

**Authors:** Mohamed Amine Benchaib, Anass Bouchnita, Vitaly Volpert, Abdelkader Makhoute

**Affiliations:** ^1^Faculté des Sciences, Université Moulay Ismail, Meknes, Morocco; ^2^Division of Scientific Computing, Department of Information Technology, Uppsala University, Uppsala, Sweden; ^3^Institut Camille Jordan, Université Lyon 1, Villeurbanne, France; ^4^INRIA Team Dracula, INRIA Lyon La Doua, Villeurbanne, France; ^5^Peoples Friendship University of Russia (RUDN University), Moscow, Russia; ^6^Marchuk Institute of Numerical Mathematics of the RAS, Moscow, Russia; ^7^Faculty of Sciences, Université Libre de Bruxelles (ULB), Brussels, Belgium

**Keywords:** PD-1/PD-L1, immune response, cancer immune interaction, hybrid modeling, multiscale modeling

## Abstract

In the advanced stages of cancers like melanoma, some of the malignant cells leave the primary tumor and infiltrate the neighboring lymph nodes (LNs). The interaction between secondary cancer and the immune response in the lymph node represents a complex process that needs to be fully understood in order to develop more effective immunotherapeutic strategies. In this process, antigen-presenting cells (APCs) approach the tumor and initiate the adaptive immune response for the corresponding antigen. They stimulate the naive CD4^+^ and CD8^+^ T lymphocytes which subsequently generate a population of helper and effector cells. On one hand, immune cells can eliminate tumor cells using cell-cell contact and by secreting apoptosis inducing cytokines. They are also able to induce their dormancy. On the other hand, the tumor cells are able to escape the immune surveillance using their immunosuppressive abilities. To study the interplay between tumor progression and the immune response, we develop two new models describing the interaction between cancer and immune cells in the lymph node. The first model consists of partial differential equations (PDEs) describing the populations of the different types of cells. The second one is a hybrid discrete-continuous model integrating the mechanical and biochemical mechanisms that define the tumor-immune interplay in the lymph node. We use the continuous model to determine the conditions of the regimes of tumor-immune interaction in the lymph node. While we use the hybrid model to elucidate the mechanisms that contribute to the development of each regime at the cellular and tissue levels. We study the dynamics of tumor growth in the absence of immune cells. Then, we consider the immune response and we quantify the effects of immunosuppression and local EGF concentration on the fate of the tumor. Numerical simulations of the two models show the existence of three possible outcomes of the tumor-immune interactions in the lymph node that coincide with the main phases of the immunoediting process: tumor elimination, equilibrium, and tumor evasion. Both models predict that the administration of EGF can promote the elimination of the secondary tumor by PD-1/PD-L1 blockade.

## 1. Introduction

Malignant cells commonly infiltrate the local, regional, and distant lymph nodes during the advanced stages of primary cancers (Dowlatshahi et al., 1997). The lymph node represents a major component of the lymphatic system and the organ where the cytotoxic T-cells (CTLs) are produced. While these cells can leave the lymph node and eliminate the tumor cells at the site of the primary tumor, they can directly eradicate the tumor cells in the lymph node upon their infiltration. The immune response begins when circulating antigen-presenting cells (APCs) capture tumor antigens and present them to the naive CD4^+^ and CD8^+^ T-cells (cross-presentation). These two cells undergo a series of asymmetric divisions culminating in the generation of mature helper and cytotoxic cells (CTL) (Chang and Reiner, 2008). The fate decision of immune cells strongly depends on the intracellular concentrations of interleukin-2 (IL-2) (Khan et al., 2015) and type I interferon (IFN) (Welsh et al., 2012). Mature CD4^+^ T-cells produce IL-2 which upregulates the maturation of T-cells while antigen-presenting cells (APCs) secrete type I IFN which increases their division. CD8^+^ eliminate the tumor cells by inducing their apoptosis through the secretion of cytokines such as Fas-Ligand (FasL). They can also inject these cytokines directly into the tumor cells during cell-cell contact.

In cancer, cells acquire mutations that affect their genetic landscape and make them proliferate excessively. One of the main pathways that are commonly altered in cancer is the EGFR/ERK pathway (Sebolt-Leopold, 2008). The final product of this pathway, the ERK protein, becomes necessary for the proliferation of the cell upon its translocation to the nucleus. The most commonly observed mutations in the MAPK/ERK pathway concern the *K-RAS, N-RAS*, and *B-RAF* genes. Such alterations can be observed especially in secondary tumors like melanoma and lung cancer (Burotto et al., 2014).

Malignant cells can resist the immune response using different strategies such as dormancy and immune suppression. Tumor cells can survive longer in the LN as they become resistant when they are in the quiescent state. There are different mechanisms governing the dormancy of the proliferating cells. First, tumor cells may enter the quiescent state when faced by a lack of available growth factors or extracellular matrix (ECM) proteins. This stress-induced dormancy is typically observed when the ERK/p38 ratio of the cell becomes low. The cell can become once again proliferating when the same ratio becomes sufficiently high. The ECM proteins, such as fibronectin and collagens, promote the activation of dormant cells due to the cross-talk between integrins, urokinase receptor (uPAR), and EGFR (Bragado et al., 2012). The complex formed by α1β5 integrins and uPAR recruits the EGFR and FAK proteins which regulates the EGFR/p38 ratio in a fibronectin-dependent manner (Barkan and Chambers, 2011). The effect of the ECM proteins on tumor dormancy is especially interesting in the case of secondary tumor development in the lymph nodes. These organs consist of distinct regions with different densities of the ECM proteins. The outer region of the lymph node contains follicles and the interfollicular zone. The ECM proteins (fibronectin, collagen, laminins) are abundant in the interfollicular area and less expressed in the follicles (Castaños-Velez et al., 1995).

Another mechanism that can cause the quiescence of the tumor cells is the immune-induced dormancy (Romero et al., 2014). In this process, effector CD8^+^ T-cells secrete type II IFN which induces and maintains the dormancy of tumor cells (Farrar et al., 1999). To escape immuno-surveillance, the malignant cells may resort to the inactivation of neighboring T-cells using immunosuppressive mechanisms. One of these most effective techniques used by tumor cells is the activation of the programmed-death 1 (PD-1) receptor present on the surface of T-cells (Zitvogel and Kroemer, 2012). After the interaction of PD-1 with its ligand PD-L1 present on the surface of tumor cells, the T-cells reduce its production of cytokines that induce apoptosis and becomes incapable of division. Therefore, the inhibition of the PD-1/PD-L1 pathway represents one of the most effective immunotherapies (Alsaab et al., 2017). Ultimately, the balance between these different mechanisms defines the three stages of immunoediting: tumor elimination, equilibrium, and tumor escape (Dunn et al., 2002).

To our knowledge, there is no mathematical model describing the interaction between secondary tumor progression and the adaptive immune response in the lymph node. Most of the existing mathematical models concern the cancer-immune dynamics at the site of the primary tumor. These models adopted different techniques and methods depending on the question of the study. The first type of developed models uses ordinary differential equations (ODEs) to simulate the population of cells over time. The simplest form of these models consists of two equations describing the competition between tumor and immune cells in a similar way to prey and predator models. In these models, tumor cells represent prey while immune cells represent predator (dOnofrio, 2005; Foryś et al., 2006). These models can be used to describe dynamics of this interaction under normal conditions (Michelson et al., 1987) and also during chemotherapy (Foryś et al., 2006; dOnofrio, 2008). Other models include more details, and therefore more equations, such as various subpopulations of immune cells (De Pillis and Radunskaya, 2003), diffusing cytokines (Dranoff, 2004; de Pillis et al., 2005), or tumor dormancy (Page and Uhr, 2005; Wilkie and Hahnfeldt, 2013). Overall, the strength of these dynamical systems is that they can be both analyzed mathematically and simulated numerically. Among the other non-spatial models, stochastic ODEs are often used to study the effect of fluctuations and noise on the interaction between tumor and immune cells (Lefever and Horsthemke, 1979). Another class of immune-cancer interaction models considers partial differential equations (PDEs) to describe the spatiotemporal aspect of this interplay. In this context, diffusion terms can be added to the previous ODEs models to capture the mobility of cells. In these models, the spatial densities of cells and concentrations of cytokines can be both described by the same type of PDEs (Bellomo et al., 2004; Matzavinos et al., 2004). To describe the interaction between immune and tumor cells in more detail, agent-based models are considered. The most commonly used agent-based modeling framework to describe this specific problem is cellular automata (CA) models (Qi et al., 1993; Mallet and De Pillis, 2006). Finally, it is possible to build more sophisticated models of tumor-immune interaction by coupling agent-based and continuous models. The resulting models allow the description of the different mechanisms affecting the behavior of the system at different scales (Gong et al., 2017). One of these models combined CA with PDEs to simulate different growth regimes that can result from the cancer-immune interplay (Mallet and De Pillis, 2006). The fate of each cell is given by a probability which depends on the local concentration of cytokines, and the number of neighboring cells. However, this study is restricted to the interaction between cancer and innate immunity. Furthermore, it does not include the dormancy of tumor cells which plays an important role in the survival and evasion of tumors. These models are stochastic by design and can be used to study the effects of immunotherapeutic treatments such as PD-1 and PD-L1 inhibitors.

This study is devoted to the mathematical modeling of the cancer-immune system interactions in the lymph node. To perform quantitative numerical simulations and ensure an accurate description of the system, we develop two models complementing each other. The first model is deterministic and uses four PDEs to describe the densities of proliferating tumor cells, dormant tumor cells, immune cells, and the concentration of a growth factor. The second model reported in this study belongs to the hybrid discrete-continuous class. In this model, cells are represented as individual objects (soft spheres) that can move, divide, differentiate, and die by apoptosis. The fate decision of each cell depends on the concentrations of intracellular proteins and extracellular cytokines described, respectively, by ODEs and PDEs. We begin with the description of tumor growth dynamics in the absence of immune surveillance. Then, we introduce the immune response and quantify the combined effect of PD-1/PD-L1 inhibition and EGF concentration on the outcome of the tumor-immune interaction in the LN. Both models confirm the existence of the three regimes characterizing the immunoediting process: tumor elimination, equilibrium, and tumor evasion. Furthermore, they reveal that combined anti-PD-1/PD-L1 therapy with growth factors can be administered in order to eradicate the tumor.

## 2. Mathematical Modeling of the Tumor-Immune Interaction in the Lymph Node

**Figure 1:**
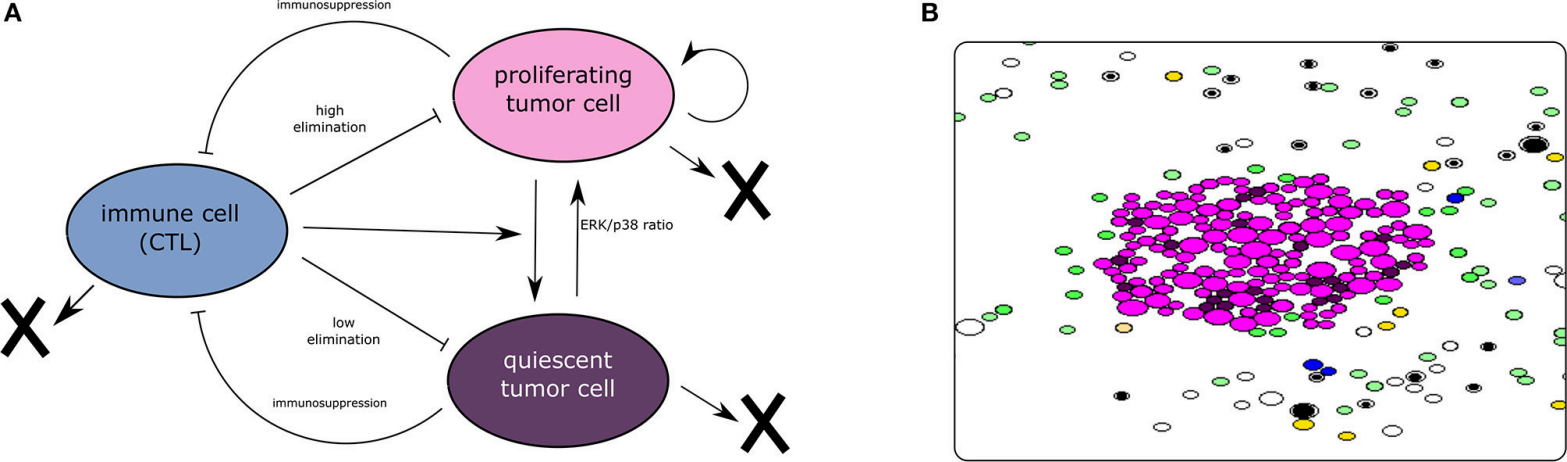
**(A)** The interaction between tumor and immune cells. Tumor cells enter dormancy when the ERK/p38 ratio is low. They become proliferating again when the same ratio becomes high. Cytotoxic immune cells can induce the dormancy of tumor cells. They can also eliminate these cells by secreting apoptosis-inducing cytokines. On the other hand, tumor cells can disable the CTLs using their immunosuppressive abilities. **(B)** A screenshot of a simulation using the hybrid model showing the proliferating and dormant tumor cells (magenta and brown, respectively), the APCs (green), the naive T-cells (white and black), the helper CD4^+^ T-cells (yellow), and the effector CD8^+^ T-cells (blue). The concentrations of extracellular cytokines are not shown.

To capture the dynamics of tumor-immune interaction, we develop two models describing this complex process. The first model adopts a population dynamics approach and uses PDEs to describe the densities of different types of cells. The model is deterministic and computationally cheap which makes it appropriate for quantitative studies. The second model is a hybrid-discrete continuous model where cells are represented as individual objects. These cells can move, auto-renew, differentiate, or die by apoptosis. Their fate is regulated by the concentration of intracellular and extracellular proteins described by ODEs and PDEs. This multiscale model is complex and computationally expensive. However, it is also more realistic as it integrates the most important mechanisms regulating the dynamics of cells. Several assumptions were considered during the development of the two models. First, we consider that the dormancy of tumor cells is mediated exclusively by the lack of EGF and the exposition to type II IFN, which is secreted by CD8^+^ T-cells. Others mechanisms that induce the dormancy of tumor cells are not included in the present model. Second, the model is restricted to the interplay between the tumor and the adaptive immune response in the lymph node. Therefore, the interaction with the innate immune response is not captured in the two models. We have represented the main interactions characterizing the models and provided a screenshot of the hybrid discrete-continuous one in Figure 1.

### 2.1. Continuous Model of the Spatiotemporal Dynamics of Tumor-Immune Interaction

After their infiltration to the lymph node, tumor cells find themselves in the direct contact with the immune cells. We propose the following population dynamics model describing the interaction between malignant cells and immune cells in the lymph node. Let us consider the spatial variables *x* and *y* as well as the temporal variable *t*, we simulate the evolution of the epidermal growth factor (EGF, *e*_*g*_(*x, y, t*)) and three populations of cells: proliferationg tumor cells (*c*_*p*_(*x, y, t*)), quiescent tumor cells (*c*_*q*_(*x, y, t*)), and immune effector cells (*i*(*x, y, t*)). The model is solved in a 2D computational domain representing the lymph node. It studies the interactions that exist between secondary cancer and immune cells in the LN without considering explicitly the underlying biological mechanisms. We begin with the equation describing the concentration of the epidermal growth factor in the lymph node:

(1) $$\begin{array}{l} \frac{\partial e_{g}}{\partial t}=D_{1}\Delta e_{g}-k_{1}\left(c_{p}+c_{q}\right)e_{g}-k_{2}e_{g}, \end{array}$$

where the first term in the right-hand side of this equation describes EGF diffusion, the second term represents EGF consumption by tumor cells, and the third term describes its degradation. Next, we describe the density of proliferating tumor cells:

(2) $$\begin{array}{l} \frac{\partial c_{p}}{\partial t}=D_{2}\Delta c_{p}+k_{3}\left(e_{g}\right)c_{p}\left(1-\left(c_{p}+c_{q}\right)\right)\\ \;+\frac{k_{4}\left(e_{g}\right)c_{q}}{1+K_{4}i}-k_{5}c_{p}i-k_{6}c_{p}i-k_{7}\left(e_{g}\right)c_{p}-k_{8}c_{p}. \end{array}$$

Here, the first term in the right-hand side represents the motility of cancer cells. The second term characterizes the logistic growth of the tumor cell population which depends on the local concentration of EGF. We consider that this rate correlates linearly with the density of the growth factor and we set $k_{3}\left(e_{g}\right)=k_{3}^{*}e_{g}$, where $k_{3}^{*}$ is a positive constant. The third term represents the activation of dormant tumor cells which is also promoted by *e*_*g*_. Similarly to the *k*_3_(*e*_*g*_), we set $k_{4}\left(e_{g}\right)=k_{4}^{*}e_{g}$. This activation can also be inhibited by effector immune cells whose density is denoted by *i*. The fourth and fifth terms describe the elimination and the induced dormancy by the immune cells, respectively. Here we consider that the cytotoxic cells directly induce the dormancy of cells by secreting type II IFN (Katsoulidis et al., 2005). The sixth term represent the natural dormancy while the last term represents cell apoptosis. Prolfierating cells enter the quiescent phase when there is a lack of EGF. Thus, we consider a negative correlation between the rate of dormancy and the local concentration of EGF $k_{7}\left(e_{g}\right)=k_{7}^{*}\left(1-e_{g}\right)$. Next, we describe the population of quisecent tumor cells (*c*_*q*_) as follows:

(3) $$\begin{array}{l} \frac{\partial c_{q}}{\partial t}=-\frac{k_{4}\left(e_{g}\right)c_{q}}{1+K_{4}i}+k_{6}c_{p}i+k_{7}\left(e_{g}\right)c_{p}-k_{9}c_{q}, \end{array}$$

where the term $-\frac{k_{4}\left(e_{g}\right)c_{q}}{1+K_{4}i}$ describes the activation of dormant cells. The terms *k*_6_*c*_*p*_ and *k*_7_(*e*_*g*_)*c*_*p*_ describe induced and normal dormancies, respectively. The last term represents cell apoptosis. We suppose that dormant cells are more resistant to elimination by immune cells and live much longer than proliferating cells. Therefore, the rate of apoptosis for dormant cells (*k*_9_) is taken much lower than the one for proliferating cells *k*_8_. Finally, we describe the population of cytotoxic T-cells in the lymph node as follows:

(4) $$\begin{array}{l} \frac{\partial i}{\partial t}=D_{3}\Delta i+k_{10}\left(i^{0}-i\right)\left(c_{p}+c_{q}\right)-k_{11}i\left(c_{p}+c_{q}\right)-k_{12}i. \end{array}$$

As before, we describe cell motion with a diffusion term. The second term in the right-hand side of this equation represents the activation of naive T-cell lymphocytes by tumor antigens. The third term describes the elimination of immune cells by immunosuppression. The rate of immunosuppression depends on the PD-L1 expression of tumor cells. We set $k_{11}=k_{11}^{*}K_{supp}$ where *K*_*supp*_ is the level of PD-L1 expression on the surface of tumor cells. The last term corresponds to cell apoptosis.

We consider a square computational domain of 25 *mm* × 25 *mm* which corresponds approximately to the maximum size reached by enlarged lymph nodes. Proliferating cells are initially located inside a circular domain (*c*_*p*0_(*x, y*) = 1 for $\sqrt{x^{2}+y^{2}}<75\mu m$) for all the simulations. We set the value of *e*_*g*_ to be constant as an initial condition for the concentration of EGF and we prescribe the same value as the Dirichlet boundary condition at all boundaries (*e*_*g*_ = *e*_*g*0_). We use the zero-flux condition at all boundaries for the populations of tumor cells and immune cells ($\frac{\partial c_{p}}{\partial n}=0$, $\frac{\partial c_{q}}{\partial n}=0$, and $\frac{\partial i}{\partial n}=0$). The finite difference method was used for the numerical implementation of the system. The values of parameters are provided in Table A1.

### 2.2. A Hybrid Discrete-Continuous Model for Multiscale Modeling of Tumor Growth in the Lymph Node

Cancer-immune interaction is based on several mechanisms affecting cells at different scales. Here, we formulate a discrete-continuous multiscale model to describe the interaction between cancer cells and immune cells in the lymph node. We have previously used hybrid models to study various physiological systems such as erythropoiesis (Eymard et al., 2015; Bouchnita et al., 2016b), multiple myeloma (Bouchnita et al., 2016a, 2017a), the immune response (Bouchnita et al., 2017b), and HIV infection (Bouchnita et al., 2017c). The hybrid model is based on some hypotheses. The regulation of tumor cells is assumed to depend solely on the EGFR/ERK, p38, and Fas signaling pathways. Other pathways such as TGF-β and PI3K-Akt are not considered in the present model. To properly present this new model, we divide it into two submodels, one for the immune response and the other one for the tumor development. Let us begin with the description of the displacement of individual cells because all cells are subject to the same mechanical laws of motion.

#### 2.2.1. Cell Motion

Each cell is characterized by the coordinates of its center *x*_*i*_ as well as by its radius. While immune cells are supposed to move randomly in the computational domain, tumor cells do not move unless they are pushed by the surrounding cells. In the process of cell division, cells increase their radius and push the surrounding cells. Each cell consists of a compressible part corresponding to the cytoplasm and an incopressible part corresponding to the nucleus. We consider a repulsive force between each two cells when the distance between their centers *h*_*ij*_ is lower than the sum of their radii *r*_1_ + *r*_2_. The motion of each cell is described by Newton's second law:

(5) $$\begin{array}{l} m{\ddot{x}}_{i}+\mu {\dot{x}}_{i}-\underset{j\neq i}{\sum }f_{ij}-F_{i}^{r}=0, \end{array}$$

where *m* is the mass of the particle, μ is the friction factor due to contact with the surrounding medium. $F_{i}^{r}$ denotes a random force applied only to the immune cells. The repulsive force between two cells is given by the formula:

$$\begin{array}{l} f_{ij}=\{\begin{array}{ccc} K\frac{h_{0}-h_{ij}}{h_{ij}-\left(h_{0}-h_{1}\right)} & , & h_{0}-h_{i}<h_{ij}<h_{0}\\ 0 & , & h_{ij}\geq h_{0} \end{array}, \end{array}$$

where *h*_*ij*_ is the distance between the centers of the two cells *i* and *j*, *h*_0_ is the sum of their radii, *K* is a positive parameter and *h*_1_ is the sum of the incompressible parts of the two cell. The force between the cells tends to infinity if *h*_*ij*_ decreases to *h*_0_ − *h*_1_.

#### 2.2.2. The Immune Response

We adapt the previously developed model of adaptive immune response (Bouchnita et al., 2017b,c) to the specific case of tumor growth in the lymph node. The model includes different types of immune cells such as APCs, naive and mature T lymphocytes.

##### Cell division and differentiation

Every 20 h of physical time, APCs and T-cells enter the computational domain around the tumor with given proportions when there is an available space. APCs capture tumor antigens as they become sufficiently close to a tumor cells. Then, they begin secreting type I IFN which promotes the differentiation of T-cells. They also present tumor antigens to naive T-cell receptors (TCRs) and induce the asymmetric divisions of T-cells. In this process, the distant daughter cell remains undifferentiated, the proximal daughter cell becomes differentiated. We consider two levels of maturation of CD4^+^ T-cells and three levels for CD8^+^ T-cells. As they reach the last maturation stage, the CD4^+^ T-cells become helper cells and start secreting IL-2. The CD8^+^ T-cells develop into cytotoxic T-cells that can kill the tumor cells either by cell-cell contact or by secreting FasL. They can also induce the dormancy of tumor cells by secreting type II IFN. Differentiated CD8^+^ and CD4^+^ T-cells can die by apoptosis or by immunosuppression.

We suppose that cells start increasing their radii as they reach half of their life cycle. If the cell divides, then two daughter cells will appear. The direction of the axis connecting their two centers is chosen randomly between 0 to 2π. The cell cycle duration for each cell is considered to be 18 h with stochastic perturbation uniformly distributed between −3 and 3 h.

##### Intracellular regulation

Activated CD4^+^ and CD8^+^ T-cell lymphocytes can only survive when there is a sufficient amount of signaling via their IL-2 and type IFN receptors. This signaling depends on the concentration of these two cytokines in the proximity of the corresponding receptors. To describe the intracellular dynamics of these two molecules, we use ODEs that depend on the value of the extracellular cytokines at the vicinity of the cell.

Let us begin with the IL-2 dependent regulatory signal dynamics in individual cells. We can describe it by the following equation:

(6) $$\begin{array}{l} \frac{dI_{i}}{dt}=\frac{\alpha_{1}}{n_{T}}I_{e}\left(x_{i},t\right)-d_{1}I_{i}. \end{array}$$

Here *I*_*i*_ is the intracellular concentration of signaling molecules accumulated as a consequence of IL-2 signals transmitted through transmembrane receptor IL2R downstream the signaling pathway to control the gene expression in the *i*th cell. The first term in the right-hand side of this equation shows the cumulative effect of IL-2 signaling. The extracellular concentration *I*_*e*_ is taken at the center of the cell (*x*_*i*_). The second term describes the degradation of IL-2-induced signaling molecules inside the cell, *n*_*T*_ is the number of molecules internalized by T cell receptors.

Similarly, we describe the IFN-α dependent regulatory signal dynamics in individual cells as follows:

(7) $$\begin{array}{l} \frac{dC_{i}}{dt}=\frac{\alpha_{2}}{n_{T}}C_{e}\left(x_{i},t\right)-d_{2}C_{i}. \end{array}$$

Here *C*_*i*_ is the intracellular concentration of signaling molecules accumulated as a consequence of IFN-α signals transmitted through transmembrane receptor IFN-αR downstream the signaling pathway to control the gene expression in the *i*-th cell. The first term in the right-hand side of this equation shows the cumulative effect of IFN-α signaling. The extracellular concentration *C*_*e*_ is taken at the center of the cell *x*_*i*_. The second term describes the degradation of IFN-induced signaling molecules inside the cell.

Finally, we describe the activation of the cell surface receptor PD-1 as a function of the local concentration of its ligand PD-L1:

(8) $$\begin{array}{l} \frac{dPD_{i}}{dt}=\alpha_{3}PD_{e}\left(x_{i},t\right)\left(1-PD_{i}\right)-d_{3}PD_{i}. \end{array}$$

As before, the first term in the right-hand side of this equation represents the cumulative effects of PD-1 activation by PD-L1, *PD*_*e*_(*x*_*i*_, *t*) is the sum of PD-L1 expression by surrounding tumor cells. The second term describes the inactivation of the PD-1 receptors at the cell surface.

**Figure 2:**
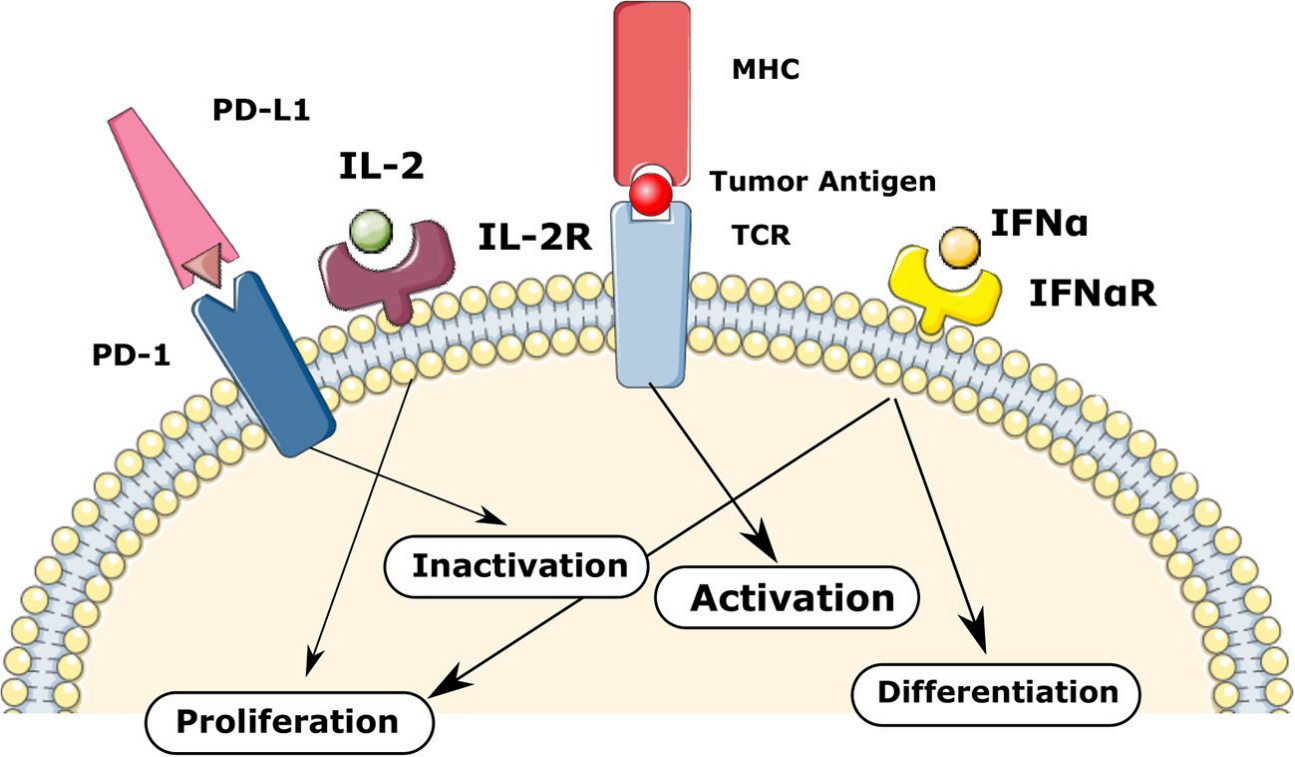
The regulation of T-cells fate in the model via TCR, IL-2, IFNa, and PD-1 mediated signaling. The balance between these signaling mechanisms provokes different fate reactions such as the activation, proliferation, differentiation, inactivation, and apoptosis.

Overall, the fate of each T-cell depends on the gene activation threshold for different signaling such as TCR, IL-2, IFNa, and PD-1 as shown in Figure 2. We consider the following decision mechanism to describe the fate regulation of activated T-cells as a function of the IL-2, type I IFN, and PD-1 signaling at different stages of the cell cycle.

C1 At the beginning of cell cycle: if the concentration of activation signals induced by type I IFN, *C*_*i*_, is greater than some critical level $C_{i}^{*}$ and that of *I*_*i*_, is smaller than the critical level $I_{i}^{*}$, then the cell will differentiate in a mature cell (Bouchnita et al., 2017b).
C2 At the end of the cell cycle: if the concentration of activation signals induced by IL-2, *I*_*i*_ is greater than some critical level $I_{i}^{*}$, then the cell will divide producing two more mature cells (Bouchnita et al., 2017b).
C3 During cell cycle: if the level of active PD-1 receptors *PD*_*i*_ exceeds a certain threshold $PD_{i}^{*}$ then the cell will be inactivated and removed from the domain.
C4 If $C_{i}<C_{i}^{*}$ at the beginning of cell cycle and $I_{i}<I_{i}^{*}$ at the end of cell cycle, then the cell will die by apoptosis and will be removed from the computational domain (Bouchnita et al., 2017b).

##### Extracellular dynamics of cytokines

The concentrations of IL-2 and type I IFN determines the differentiation and maturation of T-cells as described before. These two cytokines are produced by mature CD4^+^ T cells and active antigen-presenting cells, respectively. We use reaction-diffusion equations to describe spatial distributions of their concentrations:

(9) $$\begin{array}{l} \frac{\partial I_{e}}{\partial t}=D_{IL}\Delta I_{e}+W_{IL}-b_{1}I_{e}. \end{array}$$

Here *I*_*e*_ denotes the extracellular concentration of IL-2 and *D* is the diffusion coefficient, *W*_*IL*_ is the rate of its production by mature CD4^+^ T cells, and the last term in the right-hand side of this equation describes its consumption and degradation.

Each mature CD4^+^ T-cell secretes IL-2 in the lymph node. The production rate *W*_*IL*_ only applies at the areas of the computational domain occupied by these cells. The consumption of IL-2 is considered implicitly in the degradation term.

We suppose that antigen-presenting cells secrete type I IFN upon their activation (through direct contact with tumor antigens). The concentration of extracellular type I IFN is described by the same type of equation as IL-2:

(10) $$\begin{array}{l} \frac{\partial C_{e}}{\partial t}=D_{IFN}\Delta C_{e}+W_{IFN}-b_{2}C_{e}. \end{array}$$

As before, the production rate *W*_*IFN*_ equals ρ_*IFN*_ at the area filled by APC cells and zero otherwise. The consumption of type IFN is also considered implicitly in the degradation term.

We also consider that the cytotoxic cells (mature CD8^+^ T cells) secrete Fas-Ligand (*F*_*e*_). It is an apoptosis inducing cytokines that participate in the elimination of tumor cells. Fas-Ligand activates Fas receptors in tumor cells which induces their apoptosis. Its concentration in the extracellular matrix is described by the following equation:

(11) $$\begin{array}{l} \frac{\partial F_{e}}{\partial t}=D_{F_{L}}\Delta F_{e}+W_{F_{L}}-b_{3}F_{L}. \end{array}$$

We impose initial and boundary conditions for Equations (9–11).

#### 2.2.3. Tumor Growth

**Figure 3:**
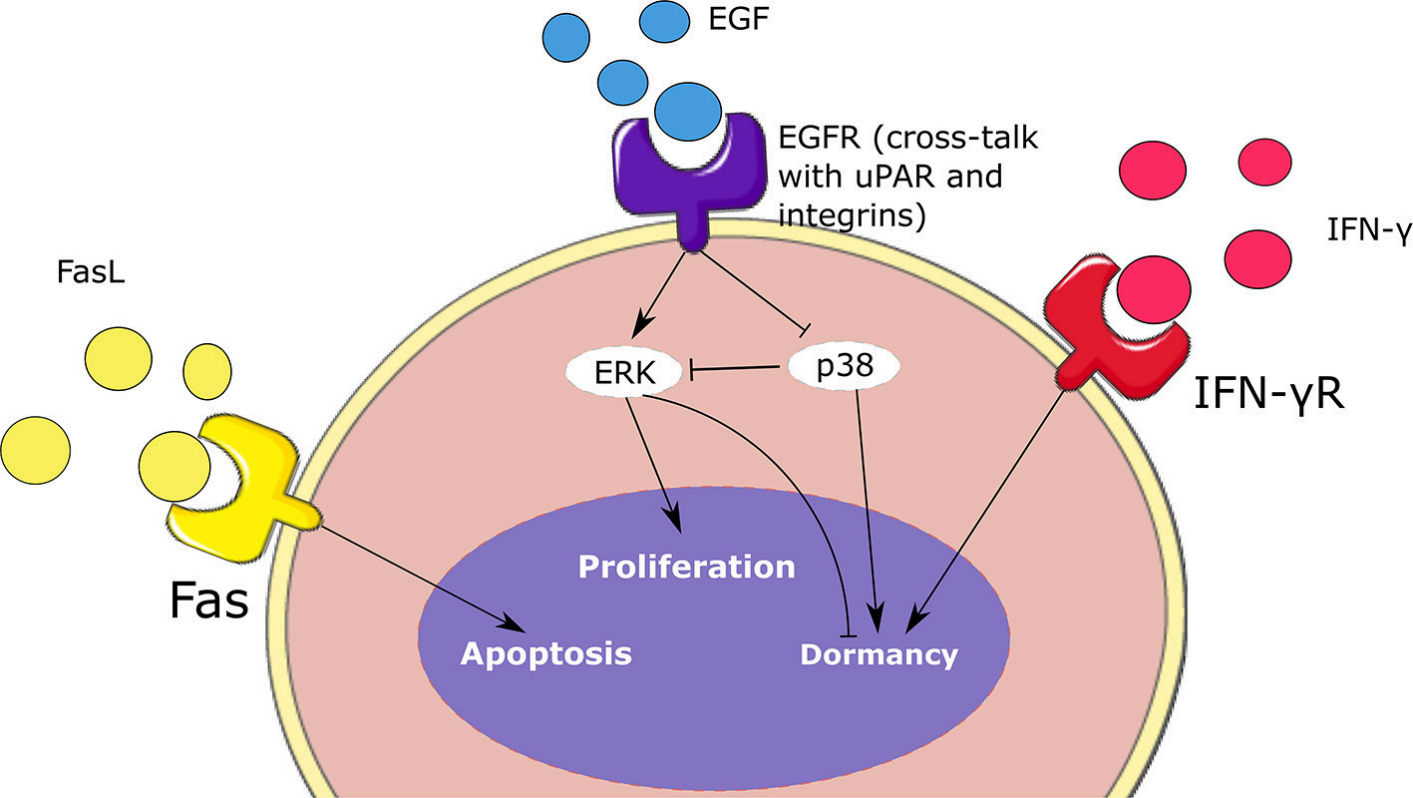
Schematic representation of the intracellular regulation of tumor cells. Cell fate depends on the concentrations of ERK, p38, type II IFN, and Fas. These signaling mechanisms contribute to the balance between the proliferation, dormancy, and apoptosis of malignant cells.

We present here the model describing secondary tumor development in the lymph node. The model includes two subtypes of malignant cells: proliferating and quiescent cells. The former can proliferate while the latter are more resistant to tumor elimination. A simplified representation of the considered intracellular regulation of tumor cells is provided in Figure 3. Each proliferating tumor cell can have three possible fates: proliferation, quiescence, and apoptosis. We consider that the tumor progression is mainly driven by the EGFR/ERK pathway because it is one of the most altered in secondary tumors (Burotto et al., 2014). These alterations are caused by gene mutations that upregulate the expression of ERK and cause the proliferation of the tumor cell. The most common mutations that affect this pathway are acquired by the *K-RAS, N-RAS*, and *B-RAF* genes. As before for the immune cells, we describe the intracellular and extracellular mechanisms regulating the fate of cancer cells.

##### Intracellular regulation

We describe the intracellular concentration of three intracellular proteins: ERK (*E*_*i*_), p38 (*P*_*i*_), and Fas (*F*_*i*_). ERK is the final product of the EGFR/ERK pathway. This signaling pathway is stimulated when the epidermal growth factor receptors present at the cell surface are activated. Subsequently, these receptors activate the Ras/Raf/MEK/ERK cascade which promotes cell proliferation (Li et al., 2016). To simplify the model, we only describe the intracellular concentration of the final product of this pathway ERK:

(12) $$\begin{array}{l} \frac{dE_{i}}{dt}=\beta_{1}G_{F}\left(x_{i},t\right)\left(E^{0}-E_{i}\right)-\gamma_{1}P_{i}E_{i}-d_{4}E_{i}. \end{array}$$

Here we suppose that ERK activation depends on the extracellular concentration of the epidermal growth factor (EGF) denoted by *G*_*F*_(*x*_*i*_, *t*). The second term in the right-hand side of this equation represents the inhibition of active ERK by the protein p38. The last term describes the degradation of ERK. This protein is activated by the FAK protein which is recruited during a cross-talk between EGFR signaling, uPAR, and integrins α1β5 (Barkan and Chambers, 2011). Next, we describe the concentration of another important protein called p38. The ERK/p38 ratio plays an important role in regulating the dormancy of tumor cells. We describe the intracellular concentration of p38 as follows:

(13) $$\begin{array}{l} \frac{dP_{i}}{dt}=\beta_{2}\left(G_{F}^{0}-G_{F}\left(x_{i},t\right)\right)\left(P^{0}-P_{i}\right)-d_{5}P_{i}. \end{array}$$

Here we consider that the lack of the cross-talk between EGFR, uPAR and integrins provokes the upregulation of p38 (Gao et al., 2012). The last term represents the degradation of p38. Another important protein whose signaling induce the dormancy of tumor cells is IFN-γR. We describe its intracellular concentration as follows:

(14) $$\begin{array}{l} \frac{dB_{i}}{dt}=\beta_{3}B_{e}\left(x_{i},t\right)-d_{6}B_{i}, \end{array}$$

where *B*_*e*_(*x*_*i*_, *t*) denotes the concentration of extracellular type II IFN at the center of the cell and *d*_6_ is the degradation rate.

A similar equation is used for the concentration of Fas:

(15) $$\begin{array}{l} \frac{dF_{i}}{dt}=\beta_{4}F_{e}\left(x_{i},t\right)-d_{7}F_{i}, \end{array}$$

where the *F*_*e*_(*x*_*i*_, *t*) represents the concentration of the extracellular FasL at the center of the cell and *d*_7_ is the degradation rate.

We consider the following decision mechanism for the regulation of each tumor cell:

D1 We consider the cell variable $\Phi_{i}=m\frac{P_{i}}{E_{i}}+n\frac{B_{i}}{B_{0}}$, where *m* and *n* are positive parameters and *B*_0_ is the average concentration of type II IFN in malignant cells. This variable quantifies the tendency of tumor cells to enter or to leave the quiescent state. During the G1 phase of the cell cycle, if Φ_*i*_ > 1 and the cell is proliferating then the cell enters the dormancy state. If Φ_*i*_ ≤ 1 and the cell is quiescent, the cell becomes proliferating.
D2 By the end of the G1 phase: if the concentration of ERK *E*_*i*_ is above a certain threshold $E_{i}^{*}$, the cell will start growing and doubling its size. Then it will divide into two daughter cells at the end of the cell cycle. Otherwise, if $E_{i}\leq E_{i}^{*}$, then it will die at the end of the cell cycle.
D3 If $F_{i}>F_{i}^{*}$ during the cell cycle, then the cell will die by apoptosis.

##### Extracellular regulation

ERK and p38 signaling depend on the local concentration of epidermal growth factor (EGF). We describe the normalized concentration of this growth factor as follows:

(16) $$\begin{array}{l} \frac{\partial G_{F}}{\partial t}=D_{G_{F}}\Delta G_{F}-W_{G_{F}}-b_{3}G_{F}, \end{array}$$

where *D*_*G*_*F*__ is the diffusion coefficient, *W*_*G*_*F*__ is the consumption rate by tumor cells, and *b*_3_ is the degradation rate. We prescribe the Dirichlet boundary condition:

$$G_{F}=G_{F0},$$

where *G*_*F*0_ is a positive constant.

In addition, the fate of tumor cells depends on the activation rate of type II IFN (IFN-γ) and Fas receptors on their surface. These receptors are activated upon their binding with the respective ligands. We describe the extracellular concentration of type II IFN (*B*_*e*_) as follows:

(17) $$\begin{array}{l} \frac{\partial B_{e}}{\partial t}=D_{IFN2}\Delta B_{e}-W_{IFN2}-b_{4}B_{e}, \end{array}$$

where *D*_*IFN*2_ is the diffusion coefficient, *W*_*IFN*2_ is its production rate, and *b*_4_ is the degradation rate. This protein is produced by effector CD8^+^ T-cells and contributes to the dormancy of tumor cells. Initial and boundary conditions are set to zero for equations this equation.

#### 2.2.4. Computer Implementation

**Figure 4:**
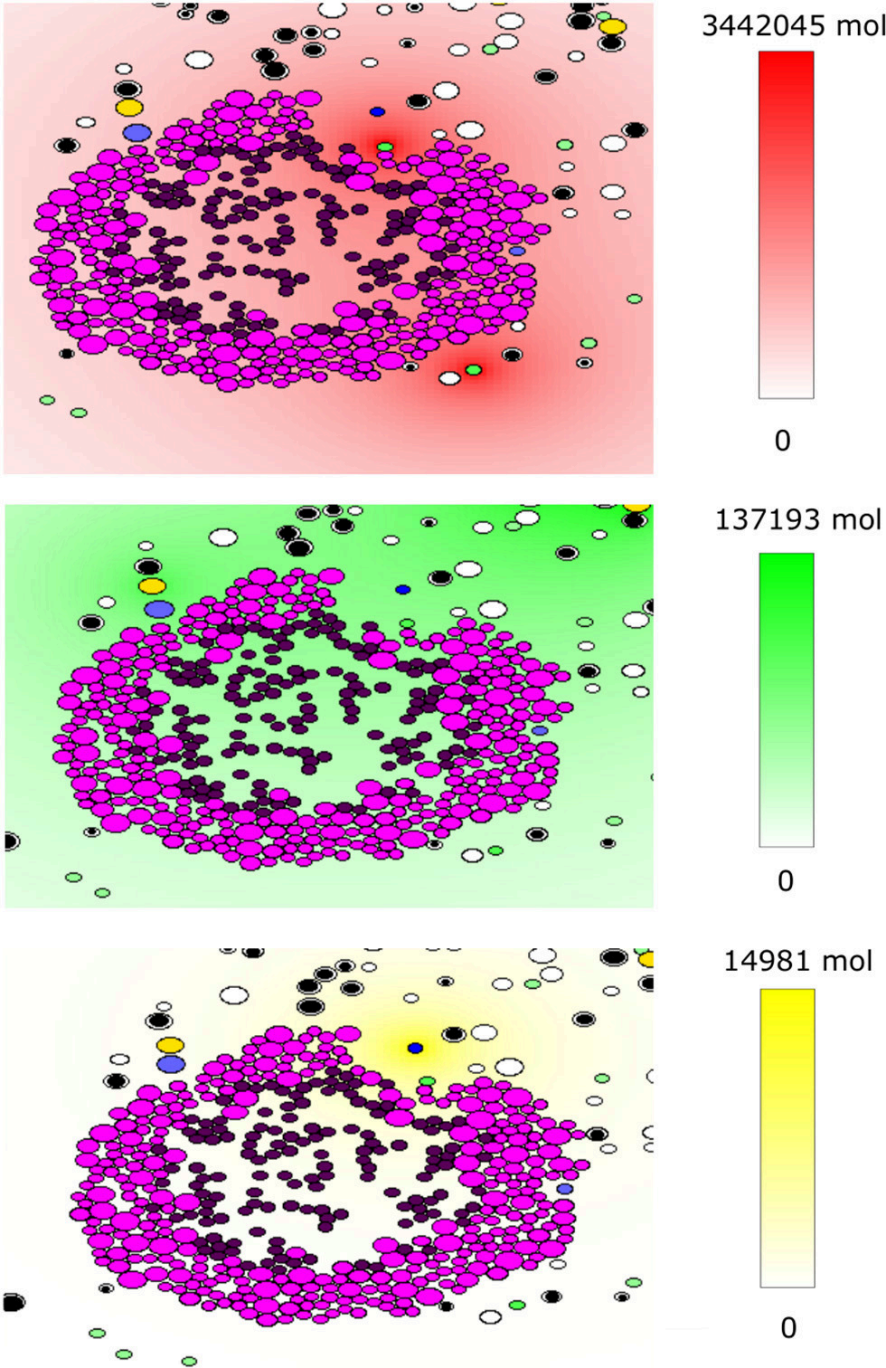
An example of a numerical simulation with the hybrid model of tumor-immune interaction in the lymph node showing the spatial distribution of the cytokines IFN-α (red, top), IL-2 (green, middle), and Fas-Ligand (yellow, bottom). Cells are shown with different colors depending on their type: magenta and purple for proliferating and dormant tumor cells, respectively, green for APCs, black and white for naive CD4^+^ T-cells and CD8^+^ T-cells, respectively, yellow for helper CD4^+^ T-cells and blue for effector CD8^+^ T-cells.

The code was written under C++ in the Object Oriented Programming style. The WxWidget library was used to visualize the simulations in real-time. The average CPU time of a numerical simulation of 3 weeks of tumor-immune interaction in the lymph node is 3 h on a computer with four cores and 6 GB of RAM. The source code is available upon request to one of the two first authors. To the opposite of the previously described continuous model, the hybrid model contains some random variables including the stochastic motion of immune cells and their introduction into the computational domain, the random direction of division, and fluctuations in the cell cycle duration. An example of numerical simulations with the hybrid model is shown in Figure 4. It shows a growing tumor surrounding by immune cells in the lymph node. The distributions of three cytokines are shown. The values of parameters are given in Table A2.

## 3. Results

### 3.1. Model Validation

We begin by comparing the output of the continuous model with the available data. Experimental results describing the effects of PD-L1 on tumor evasion (Juneja et al., 2017) were used to calibrate the model. Although these experiments describe the development of primary colorectal adenocarcinoma outside of the lymph node, they are still useful for our study because mutated MC38 cells commonly migrate to neighboring lymph nodes in the advanced stages of the disease. The aim of these experiments was to elucidate the effect of PD-1 blockade on tumor evasion. To achieve this, PD-1 and PD-L1 blocking antibodies were administrated to mice with MC38. The study shows that the immunosuppressive effect of the PD-1/PD-L1 pathway is sufficient to cause the evasion of the tumor.

**Figure 5:**
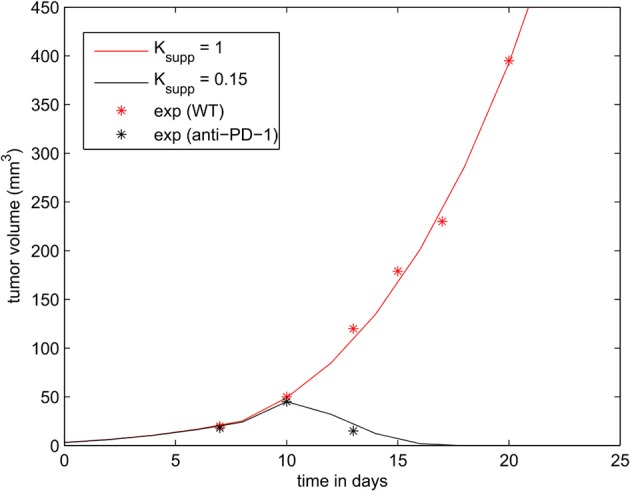
The effect of PD-1 blockade on the growth of the tumor. Results of the numerical simulations using the continuous model are compared with experimental data in Figure 1B of Juneja et al. (2017) (shown in dots).

The parameters of the model were calibrated to reproduce the experiments presented in Figure 1B of Juneja et al. (2017). The volume of the tumor was calculated using the formula $V=\frac{4}{3}\pi r^{3}$, where *r* is the radius of the tumor. The tumor growth rate ($k_{3}^{*}$) was fitted to reproduce the growth of the wild-type MC38 tumor. Then, we determined the rate of immunosuppression ($k_{11}^{*}$) in such a way that the administration of a PD-1 inhibitor corresponds to a reduction of the level of PD-1 by 85 % (*K*_*supp*_ = 0.15). With these modifications, the model is able to accurately reproduce the experimental data as shown in Figure 5.

### 3.2. Tumor Growth Dynamics in the Absence of Immune Response

**Figure 6:**
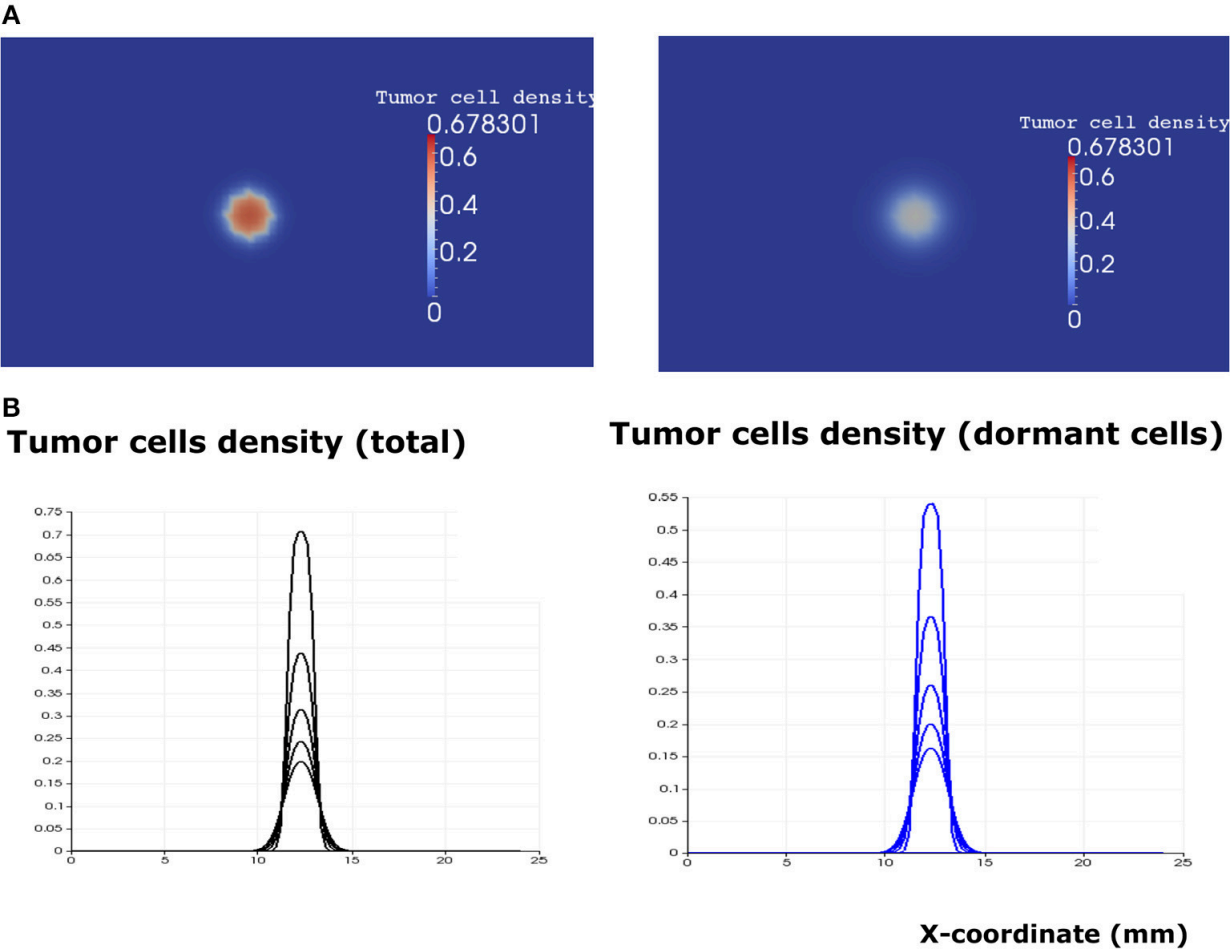
The regime of tumor elimination is observed when the value of the epidermal growth factor in the lymph node corresponds to *e*_*g*0_ = 0.3. **(A)** 2D representation of the tumor at the beginning of day 4 (left) and day 8 (right). **(B)** Plots of tumor cell densities at the cross-section at different times of the simulation (every 4 days) showing the elimination of the tumor through the apoptosis of proliferating and dormant cells.

After the calibration of the model, we proceed to study its dynamics in the absence of immune response (*i*^0^ = 0). Then the system can be reduced to three Equations (1–3). We solve them numerically for different parameter values. Since the EGFR pathway represents a possible target for cancer treatment, we quantify the effect of the EGF concentration in the lymph node (*e*_*g*0_) on the development of the tumor. Numerical simulations show the existence of two different scenarios. When the concentration of EGF is below the threshold value $e_{g0}^{*}=0.55$, the tumor does not develop and will be eliminated within few weeks depending on the available concentration of the EGF (Figure 6).

**Figure 7:**
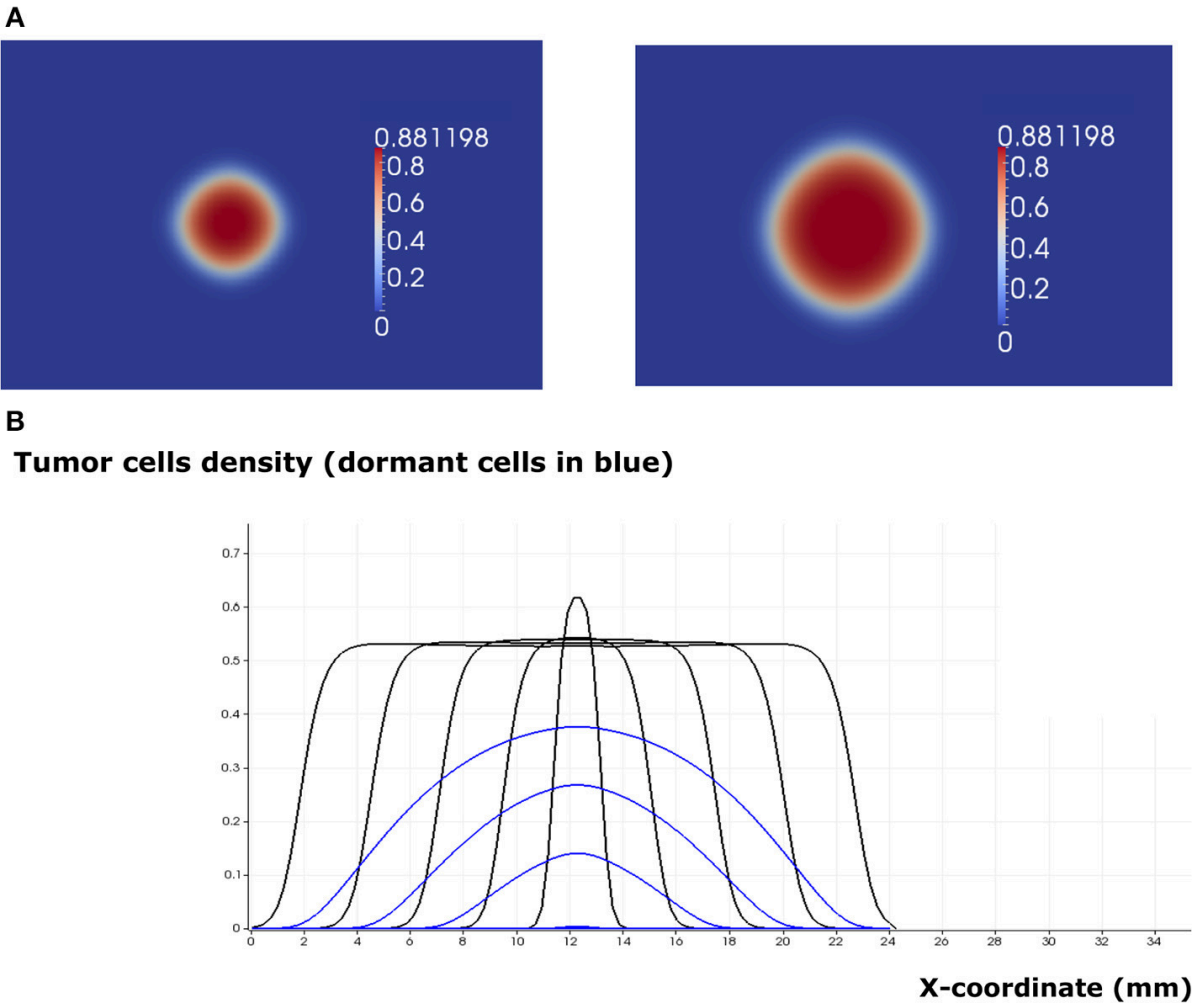
The regime of tumor evasion in the case where the value of the epidermal growth factor in the lymph node corresponds to *e*_*g*0_ = 0.8 and in the absence of immune response. **(A)** 2D representation of the tumor at the beginning of day 4 (left) and day 8 (right). **(B)** Plot of tumor cell densities at the cross section at different times of the simulation (every 4 days) showing the traveling wave solution (only for the total number of cells). The plots corresponding to the populations of dormant cells are shown in blue.

When the EGF concentration in the lymph node exceeds the threshold value $e_{g0}^{*}$, the tumor cells increase their proliferating rate. Tumor growth is exponential in the beginning and linear after some time. The tumor expands in the form of a traveling wave and invades the neighboring tissue (Figure 7). The speed of wave propagation increases as the value of EGF in the lymph node grows. We conclude that there exist two possible regimes of tumor development in the absence of immune response: elimination and evasion. It is possible to obtain these two regimes by varying other parameters of the model, for example, depending on the proliferation and apoptosis rates of tumor cells determined by their phenotypes. The rate by which tumor cells enter and leave the quiescent state also determines the regime of tumor development and can vary depending on different conditions such as hypoxia and TGF-β signaling.

**Figure 8:**
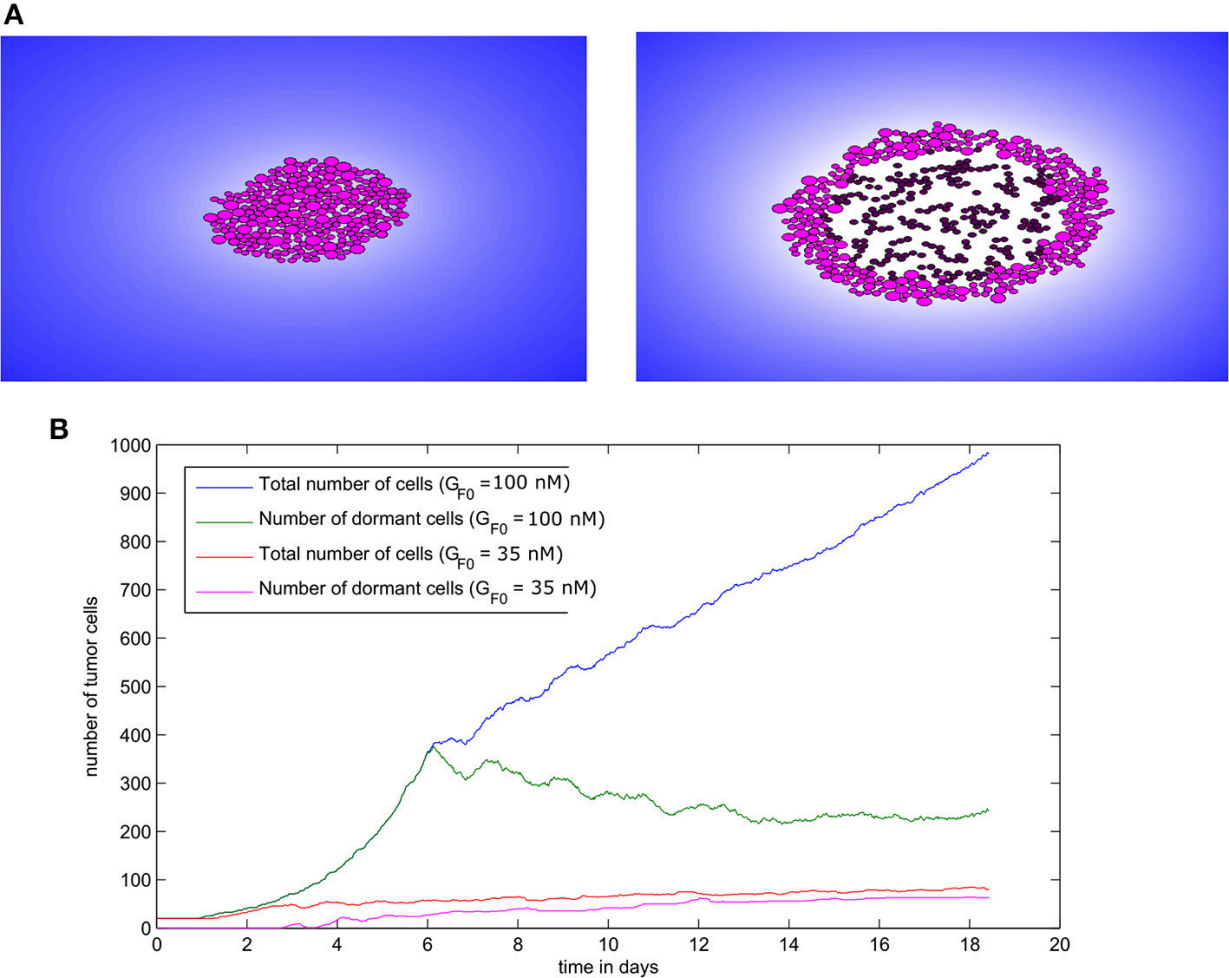
Tumor growth in the absence of immune response as simulated with the hybrid model. **(A)** Two consecutive snapshots of a numerical simulation showing tumor development in the absence of immune response for the value *G*_*F*0_ = 100*nM*. **(B)** The populations of malignant cells (including dormant ones) in the course of two numerical simulations corresponding to two values of *G*_*F*0_.

The two regimes of tumor development in the absence of immune response can also be obtained with the hybrid model. To achieve this result, we consider an initial population of 20 cells and we run numerical simulations for different values of the EGF concentration in the LN (*G*_*F*0_). When this concentration is greater than or equal to the threshold value $G_{F0}^{*}=35nM$, the tumor grows and expands in the form of a traveling wave. When it becomes sufficiently large, the consumption of EGF at the core of the tumor will induce the dormancy of cells in this area (Figure 8A). Hence, a tumor spheroid organization composed of proliferating cells in the outer region and dormant cells at the core can be observed. In the regime of tumor escape, the ratio of dormant cells to proliferating cells becomes higher as the EGF concentration decreases (Figure 8B). When the EGF level is below the threshold value $G_{F0}^{*}$, the tumor cells will either die by apoptosis or will keep switching back and forth between the dormant and proliferating state until they die.

### 3.3. The Regimes of Tumor-Immune Interaction in the Lymph Node in the Continuous Model

After studying the dynamics of tumor growth in the absence of the immune response, we now introduce the immune cells to the model (*i*^0^ = 1), and we numerically solve the system (1–4) for different values of the model parameters. Immune cells affects the dynamics of tumor development by different mechanisms. First, they can eliminate tumor cells by secreting apoptosis-inducing cytokines such as FasL. Second, they can induce the dormancy of proliferating tumor cells by secreting type II interferon (IFN-γ). Finally, they can prevent the reactivation of dormant cells with the same mechanism (Farrar et al., 1999). It is therefore important to study the outcome of the tumor-immune interplay under different conditions.

**Figure 9:**
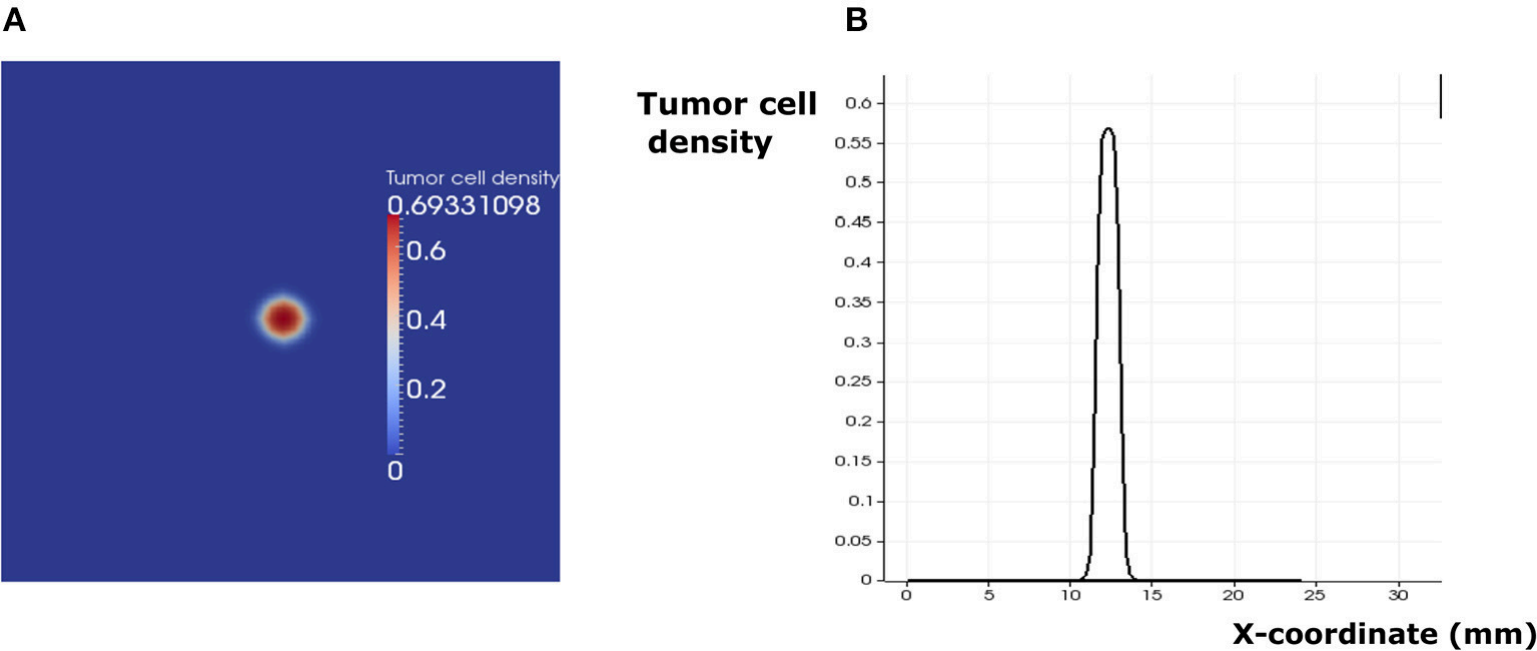
The regime of cancer immune equilibrium. **(A)** A snapshot of the solution. **(B)** Cross section plot of the stationary solution.

Depending on the parameters of the model, it is possible to observe three different regimes of tumor development. In addition to the previously described regimes that can be observed in the absence of immune response (tumor elimination and escape), one more regime can be obtained when the immune response is considered. This regime corresponds to the cancer-immune equilibrium state where the tumor and the immune cells coexist in the lymph node. In this case, immune cells cannot eradicate the tumor and they do not allow it to further develop and expand. This regime can be observed only if two conditions are fulfilled: immune cells should prevent tumor growth and the activation of dormant tumor cells. Under these two conditions, a pulse-shaped stationary solution is reached after several days of the tumor progression (Figure 9). The solution can still slowly evolve because of the low apoptosis rate of the tumor cells.

**Figure 10:**
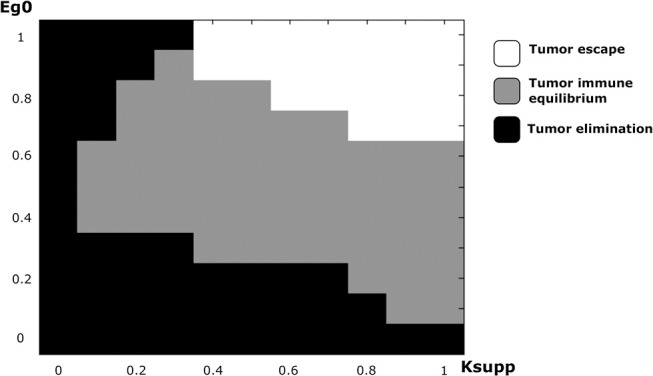
The regimes of cancer-immune interaction in the lymph node in the parameter space (*K*_*supp*_ − *e*_*g*0_).

In addition to the EGFR pathway, PD-1 and PD-L1 constitute a potential target in cancer therapy. By inhibiting their immunosuppressive abilities, tumor cells become vulnerable to immune cells and therefore can die by apoptosis during the early stages of tumor development. While it is possible to study the effect of PD-1 expression on the dynamics of the cancer-immune interaction, it is more interesting to investigate the combined effects of PD-1 (*K*_*supp*_) and EGF (*e*_*g*0_) on this process (Figure 10). Numerical simulations reveal that the equilibrium regime can only be observed for the values of *e*_*g*0_ between 0.1 and 0.9. The escape scenario can be avoided if the EGF concentration in the LN is below 0.7. The elimination of the tumor can be obtained if the immunosuppressive abilities of the tumor are very low. Interestingly, it is also possible to eradicate the tumor if the value of EGF concentration in the LN is above 0.9 and the *K*_*supp*_ value is below 0.3. Thus, the model predicts that a possible combination of PD-1/PD-L1 inhibitors with exogenous EGF can be administrated in order to overcome the drug resistance provoked by cell dormancy.

### 3.4. The Hybrid Model Reveals the Mechanism Regulating the Regimes of the Tumor-Immune Interplay in the Lymph Node

**Figure 11:**
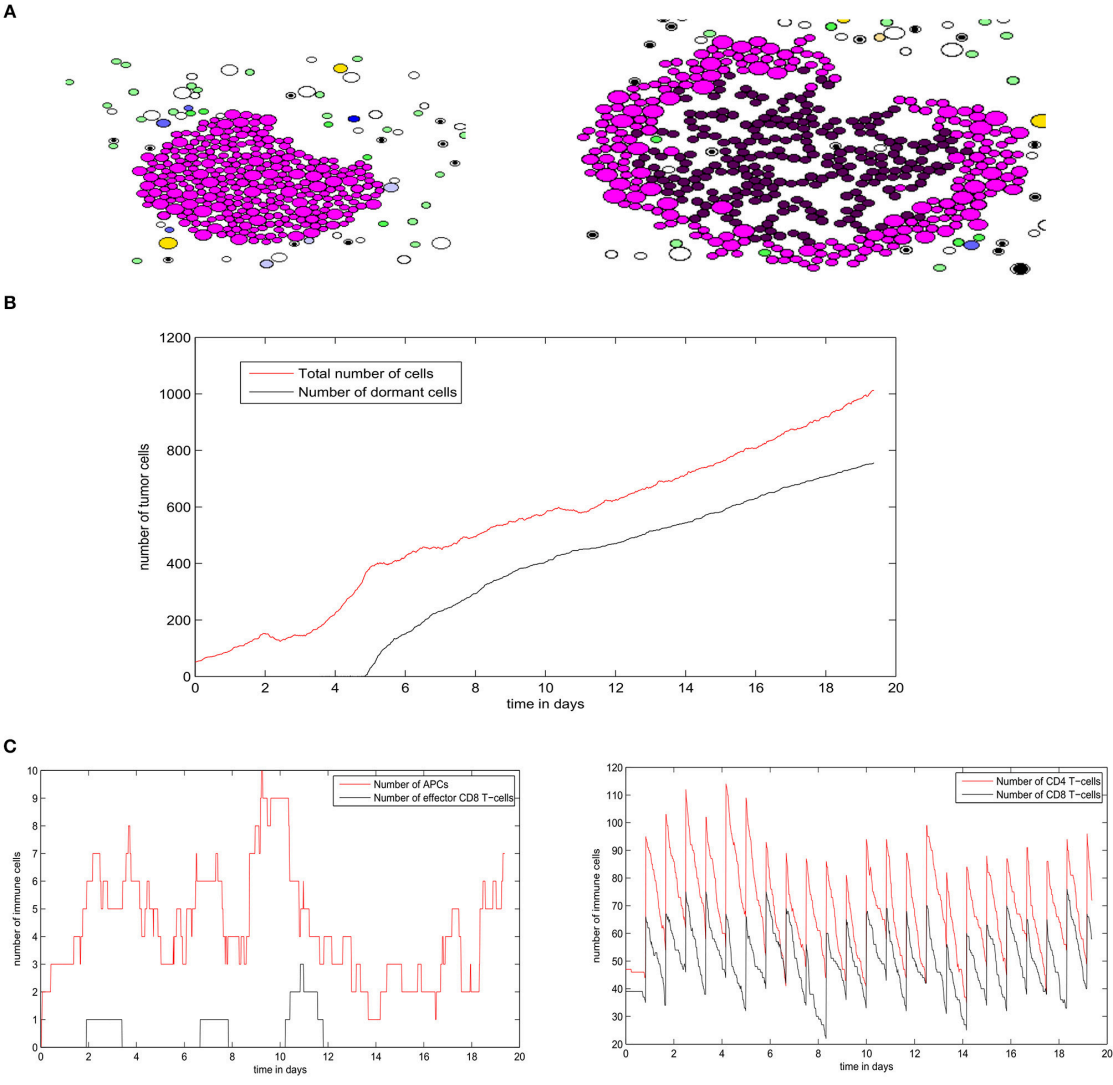
A numerical simulation using the hybrid model showing the regime of tumor escape. **(A)** Two consecutive snapshots of a numerical simulation showing the irregular shape of the growing tumor. **(B)** The populations of tumor cells in the course of the simulation. **(C)** The numbers of different immune cells over time during the development of the secondary tumor.

To elucidate the mechanisms underlying the interaction between secondary tumors and the immune response in the lymph node, we use the hybrid model to conduct numerical simulations under various conditions. We begin by studying the regime of tumor escape in the case where the concentration of EGF in the LN is equal to *G*_*F*0_ = 100*nM* and we introduce 50 tumor cells to the computational domain as an initial condition. At the beginning of the simulation, the tumor expands rapidly, and the tumor cells use their immunosuppressive abilities to escape the surveillance of the immune cells. However, some parts of the tumor are still eliminated due to the secretion of FasL by the surviving CD8^+^ T-cells. As a result, the tumor grows faster toward the directions where the presence of effector T-cells is low and the shape of the tumor becomes irregular and nonspherical (Figure 11A). The ratio of dormant to proliferating cells becomes higher in the presence of immune cells than in their absence (Figure 11B). This can be explained by the induced dormancy or elimination of a part of the proliferating cells by the effector CD8^+^ T-cells and released cytokines. Still, the tumor manages to dismantle the immune response and to develop using the immunosuppressive capacity of malignant cells. This is reflected by the low number of effector CD8^+^ T-cells despite the high number of antigen-bearing APCs as shown in Figure 11C.

**Figure 12:**
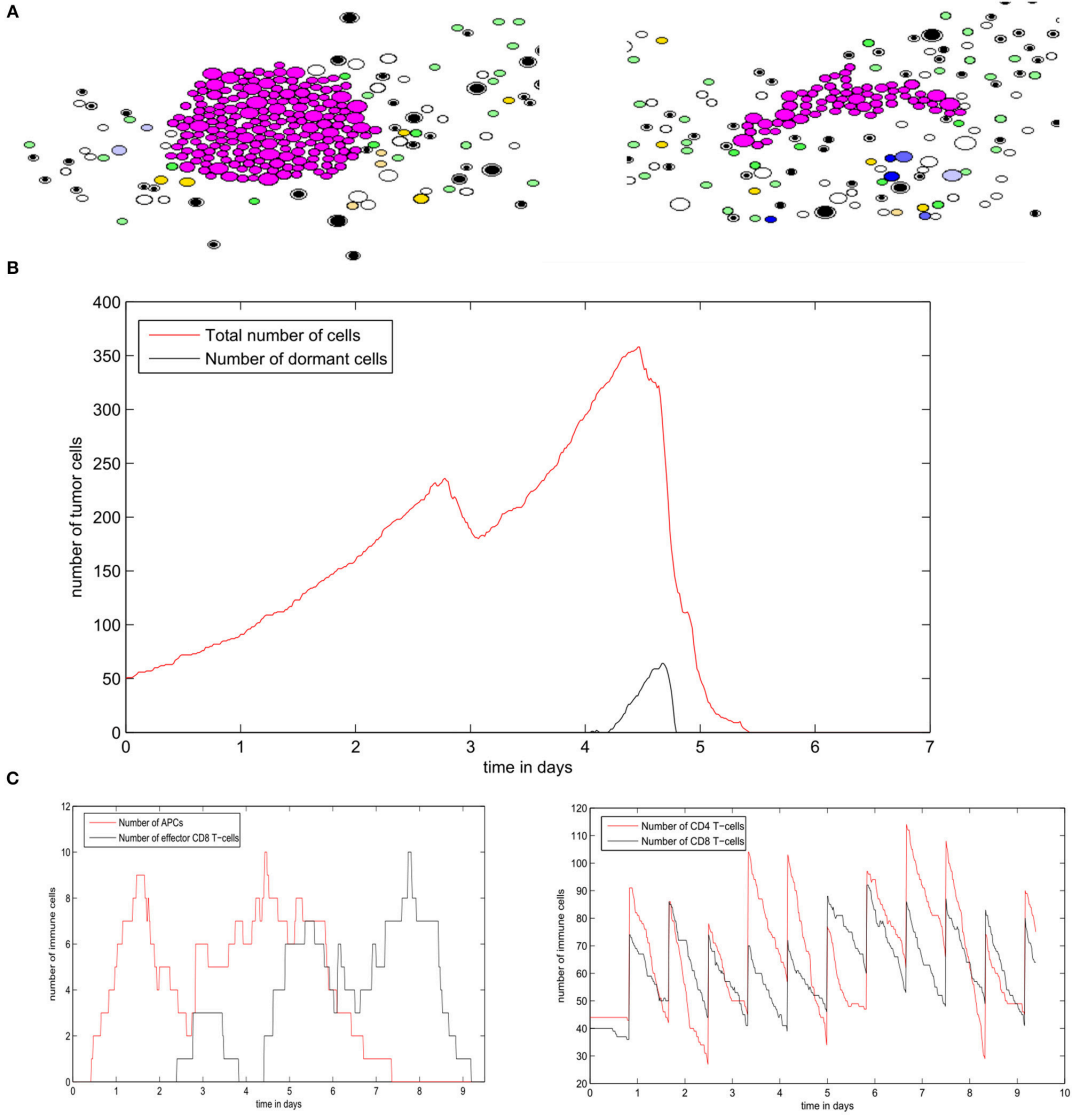
Results of a numerical simulation using the hybrid model of the regime of tumor elimination caused by the inhibition of immunosuppression. **(A)** Two snapshots of the simulation showing the elimination of the tumor. **(B)** The populations of tumor cells during the simulation. **(C)** The populations of immune cells including antigen-bearing APCs and effector CD8^+^ T-cells (left) as well as the total number of CD4^+^ and CD8^+^ T-cells (right).

Next, we study the regime of tumor elimination by the immune response by maintaining the same settings as in the previous simulation and reducing the expression of PD-L1 per cell by decreasing *PD*_*e*_/cell from 0.5 to 0.02. During the first 4–5 days, the tumor grows and expands without being affected by the immune response. This period corresponds to the necessary time for the adaptive immune response to be activated following the exposure to tumor-antigens. The inhibition of immunosuppression prevents the tumor from evading the surveillance of immune cells. As a result, a relatively important number of effector CD8 T-cells will be produced and eliminate all tumor cells within several hours (Figure 12A). Effector T-cells eradicate the tumor by secreting apoptosis inducing cytokines such as FasL that diffuse in the ECM. They can also directly transfer these cytokines into tumor cells during cell-cell contact. During the whole simulation, tumor cells will be proliferating except for few cells that appeared at the beginning of the fourth day. These cells quickly leave the quiescent state because they are exposed to growth factors (Figure 12B). In the absence of immunosuppression, the number of effector CD8^+^ T-cells will become high after the first 4 days while antigen-bearing APCs will extinct after the elimination of the tumor, as revealed in Figure 12C. It is important to note that the low expression of PD-L1 by malignant cells is not sufficient to observe the elimination of the tumor. With current model settings (*PD*_*e*_/cell = 0.02), this regimes can only be observed if the EGF concentration is higher than 70 *nM* or lower than 10 *nM*. This supports the conclusion drawn from simulations of the continuous model which implies that the administration of EGF can result in the elimination of the tumor by anti-PD-1/PD-L1 agents.

**Figure 13:**
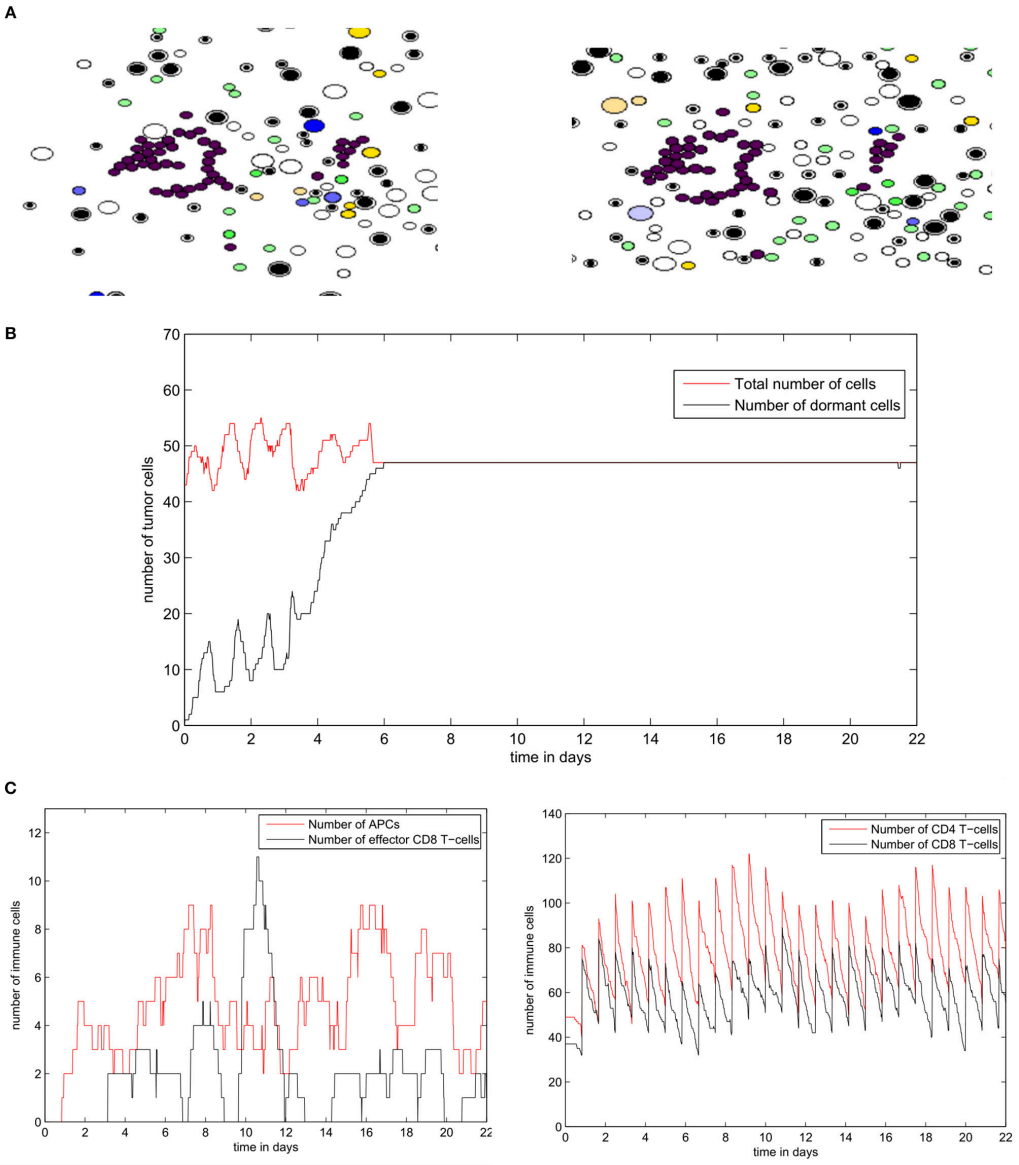
Numerical simulation of the tumor-immune equilibrium in the lymph node using the hybrid model. **(A)** Snapshots of a numerical simulation. **(B)** The population of tumor cells. **(C)** The number of immune cells during the simulation.

The regime of tumor-immune equilibrium can only be observed when the concentration of EGF in the LN is reduced. We set *G*_*F*0_ = 50*nM* and we suppose that tumor cells have a normal PD-L1 expression (*PD*_*e*_ = 0.5). In this case, the cells start dividing, and then they enter the quiescent state due to the lack of EGF and the presence of type II IFN secreted by CD8^+^ T-cells. They remain dormant for the rest of the simulation time (Figure 13A). As a result, the population of tumor cells remains constant (Figure 13B). Due to the low concentration of EGF, the cells can be maintained in a dormant state if they are constantly supplied by type II IFN. Figure 13C shows that a few numbers of effector CD8^+^ T-cells remain in proximity of the tumor during the simulation. However, these cells cannot eliminate the tumor cells because of the resistance acquired due to dormancy. Hence, they only maintain the tumor cells in a dormant state by producing type II IFN. The role of CD8^+^ T-cells in maintaining tumor cells in a dormant state was investigated in a previous *in vivo* experimental study (Farrar et al., 1999).

## 4. Discussion

This study presents two models of the tumor-immune interaction inside the lymph node. The first model uses a population dynamics approach to study the spatial dynamics of the interplay between tumor and immune cells while the second model follows a multiscale approach for a more realistic representation of the mechanisms involved in this process. The continuous model was calibrated in order to reproduce the results of an experimental study on the effects of PD-1 in tumor evasion (Juneja et al., 2017). These experiments describe tumor-immune interaction outside of the LN. However, they were still useful for the identification of the model parameters for several reasons. First, the growth rates of primary and secondary tumors are approximately the same (Peng et al., 2018; Zhang and Niedermann, 2018). Second, the parameters of the PDE model are kinetic constants that describe the rates of cellular processes such as division, dormancy, and apoptosis. These processes depend on the phenotype of the cell and not the location of the tumor. Finally, CD8 T-cells migrate to the site of the site of the tumor regardless if it is inside or outside the LN (Chheda et al., 2016). The hybrid model also qualitatively confirms the conclusions of this study by showing that PD-1 is sufficient to cause the evasion the evasion of the tumor. However, both models reveal that this conclusion is only valid in tissues with very low or very high concentration of EGF in the LN. Indeed, the higher the concentration of EGF in the LN, the higher the number of proliferating cells in the tumor, and therefore the more responsive the tumor will be to anti-PD-1/PD-L1 therapy. This result represents a testable hypothesis that can be considered in the design of future experimental studies and clinical trials. The two models were used in parallel to study an important question: what are the possible outcomes of the interaction between secondary cancer and the immune system in the lymph node. To investigate this point, we began by studying the dynamics of tumor growth in the absence of immune cells and we have shown, using both models, that the resulting tumor will have a spheroid shape with two layers: a proliferating zone in the outside layer and a quiescent zone in the inside layer. This agrees with the previous *in vivo, in vitro*, and *in silico* studies (Weiswald et al., 2015; Sant and Johnston, 2017). This spheroid organization depends on the access of tumor cells to growth factors. In both continuous and hybrid models, we have demonstrated the existence of a threshold value for the EGF concentration in the LN that separates the regimes of tumor evasion and tumor elimination.

Next, we have introduced immune cells in the model and studied their interaction with the growing tumor. In particular, we have studied the role of the immuno-suppressive mechanism in the dynamics of tumor progression in tissues with different EGF concentrations. We have shown that immunosuppression plays an important role in the evasion of the tumor from immuno-surveillance. Furthermore, we observed three different regimes of tumor growth in the lymph node using both the continuous and hybrid models. These regimes consist of the elimination of the tumor, the cancer-immune equilibrium, and the evasion of the tumor. They are already reported in the biomedical literature, and they constitute the three main phases of the immunoediting process (Dunn et al., 2004). Therefore, the parameters of the model can be tuned to study the interaction between the immune response and LN metastases of other phenotypes of malignant cells in the normal conditions or during immunotherapy. Despite the differences between the two models, the same mechanisms were considered by both of them. In the continuous model, the kinetic rates of tumor dormancy and activation depend on the local concentration of EGF and the density of immune effector T-cells. In the hybrid model, these mechanisms were introduced explicitly by considering that the state of the cell depends on the ratio ERK/p38 as well as type II IFN secreted by mature CD8^+^ T-cells. Overall, the two models allowed us to determine the conditions of each regime of tumor-immune interaction. This is important for the development of personalized immunotherapeutic strategies that consider the characteristics of individual patients and the site of metastatic tumors. In the future, we will use these two models to study the efficacy and safety of the treatment regimens applied to the therapy of secondary tumors in the lymph node. We will focus especially on the combination PD-1 and PD-L1 inhibitors with other chemotherapeutic agents.

The synergy between the continuous and hybrid model was essential for a proper representation of the cancer-immune interaction in the lymph node. On one hand, the continuous model is better suited for systemic parametric studies because numerical simulations are computationally cheap. Mathematical analysis of the model will be presented in a forthcoming work. On the other hand, the hybrid model, although computationally expensive, provides a more detailed description of the various mechanisms involved in the cancer immune interaction problem. Hence, it is possible to conduct more biologically accurate studies using this model. However, this model is less robust because it contains numerous sources of stochasticity such as low number of cells, their random motion, and the duration of the cell cycle. Thus, it is possible to observe two different regimes of tumor growth when repeating the same simulation twice and with the same parameter set.

In general, there exist few hypotheses that were considered while formulating the model. The interpretation of the obtained results was possible because of these considered a priori assumptions. First, the interaction between secondary tumors and the innate immunity in the LN was not studied in the present work. Tumor cells can evade the surveillance of natural killer (NK) cells by reducing the expression of major histocompatibility complex (MHC) class I molecules. Other considered assumptions include the simplifications that were introduced to the signaling pathways responsible for inducing the dormancy of tumor cells such as the PI3K-AKT cascade. In our models, we assume that only EGFR/ERK, p38 and Fas signaling pathways that are disturbed for tumor cells. Thus, we restrict the intracellular regulation of tumor cells to these three pathways. Furthermore, we do not include the other mechanisms that induce the dormancy and reactivation of tumor cells such as stress, hypoxia, angiogenesis, and other microenvironmental factors. Despite the recent advances in our understanding of the factors inducing the dormancy of cells, the decision mechanisms by which cells enter and leave the quiescent state remain poorly understood. Therefore, more experimental and modeling studies should focus on this particular area of research.

## Author Contributions

All authors contributed to the design of the study and the writing of the manuscript. AB and VV developed the mathematical models. MB and AB conducted the numerical simulations. All authors read and approved the final manuscript.

### Conflict of Interest Statement

The authors declare that the research was conducted in the absence of any commercial or financial relationships that could be construed as a potential conflict of interest.

## Appendix

In this section, we present the numerical values that were assigned to the parameters of the two model. The values of the continuous model were identified by fitting the results with experimental data Juneja et al. (2017) (Table A1). For the hybrid model, the values of the immune response component of the model were taken from previous works Bouchnita et al. (2017b,c). The rest of parameters were fitted in order to obtain the desirable behavior of the model. Their values are given in Table A2.

**Table A1:** The values of parameters used in numerical simulations with the continuous model.

**Parameter**	**Value**	**Unit**	**Description**
*dx*	0.25	*mm*	Spatial step
*dt*	0.001	d	Time step
*D*_1_	0.86	*mm*^2^d^−1^	Diffusion coefficient of EGF
*D*_2_	0.017	*mm*^2^d^−1^	Diffusion coefficient of proliferative tumor cells
*D*_3_	0.05	*mm*^2^d^−1^	Diffusion coefficient of CTL immune cells
*k*_1_	0.01	*d*^−1^	EGF consumption by tumor cells
*k*_2_	0.005	*d*^−1^	EGF degradation
*k*_3_	1.1	*d*^−1^	The growth rate of proliferative tumor cells
*k*_4_	0.1	*d*^−1^	The reactivation rate of dormant cells
*K*_4_	100		Inhibition constant of dormant cells reactivation by CTL
*k*_5_	2	*d*^−1^	The elimination rate of proliferative cells by CTLs
*k*_6_	0.8	*d*^−1^	The rate of immune-induced dormancy by CTLs
*k*_7_	0.5	*d*^−1^	The dormancy rate of malignant cells
*k*_8_	0.3	*d*^−1^	The apoptosis rate of proliferative cells
*k*_9_	0.000001	*d*^−1^	The apoptosis rate of dormant cells
*k*_10_	1	*d*^−1^	The rate of CTLs activation by tumor cells
*k*_11_	varied	*d*^−1^	The rate of immunosuppression

**Table A2:** Values of parameters used in simulations with the hybrid model. δ is an arbitrary length unit.

**Parameter**	**Value**	**Unit**	**Description**
*dt*	0.02	*min*	Time step
*D*_*GF*_	0.0011	δ^2^.*h*^−1^	Diffusion coefficient of EGF
*D*_*IFN*2_	0.0011	δ^2^.*h*^−1^	Diffusion coefficient of type II IFN
α_3_	0.6	Normalized	Rate of PD-1 activation by PD-L1
β_1_	1.8	*nM*^−1^.*h*^−1^	ERK activation by EGFR-uPAR-Integrins cross-talk
β_2_	3	*nM*^−1^.*h*^−1^	p38 activation by EGFR-uPAR-Integrins cross-talk
β_3_	0.024	*mol*^−1^*h*^−1^	Type II IFN activation
β_4_	0.3	*mol*^−1^*h*^−1^	Fas activation rate by FasL
γ_1_	0.2	*h*^−1^	ERK inhibition by p38
*d*_3_	4.8	*h*^−1^	Inactivation rate of PD-1
*d*_4_	0.6	*h*^−1^	Degradation rate of intracellular ERK
*d*_5_	1	*h*^−1^	Degradation rate of intracellular p38
*d*_6_	0.0228	*h*^−1^	degradation rate of type II IFN
*d*_7_	0.3	*h*^−1^	Degradation rate of Fas
*E*^0^	1	Normalized	The capcity of ERK in each cell
*P*^0^	1	Normalized	The capcity of p38 in each cell
$PD_{i}^{*}$	1	Normalized	Threshold of T-cell inactivation by PD-1/PD-L1 stimulation
$E_{i}^{*}$	0.6	Normalized	Threshold of ERK that induces proliferation
$F_{i}^{*}$	6000	Molecules	Threshold of Fas that induces cell apoptosis
*m*	0.6	Normalized	Natural dormancy coefficient
*n*	0.4	Normalized	Immune-induced dormancy coefficient
*B*^0^	1000	Molecules	The average concentration of type II IFN per cell
